# A case with assessment of cardiac–coronary interactions with transthoracic echocardiography before and after transcatheter aortic valve replacement

**DOI:** 10.1093/ehjcr/ytae037

**Published:** 2024-01-22

**Authors:** Wataru Suzuki, Masanobu Fujimoto, Hirohiko Ando, Tetsuya Amano

**Affiliations:** Department of Cardiology, Aichi Medical University, 1-1, Yazakokarimata, Nagakute, Aichi 480-1195, Japan; Department of Cardiology, Aichi Medical University, 1-1, Yazakokarimata, Nagakute, Aichi 480-1195, Japan; Department of Cardiology, Aichi Medical University, 1-1, Yazakokarimata, Nagakute, Aichi 480-1195, Japan; Department of Cardiology, Aichi Medical University, 1-1, Yazakokarimata, Nagakute, Aichi 480-1195, Japan

## Case description

In patients with aortic stenosis (AS), increased systolic wall stress due to increased afterload reduces systolic coronary flow, creating abnormal pattern of coronary flow.^1^ Aortic stenosis exhibits a complex pathophysiology involving cardiac–coronary interactions.^2^

**Figure 1:**
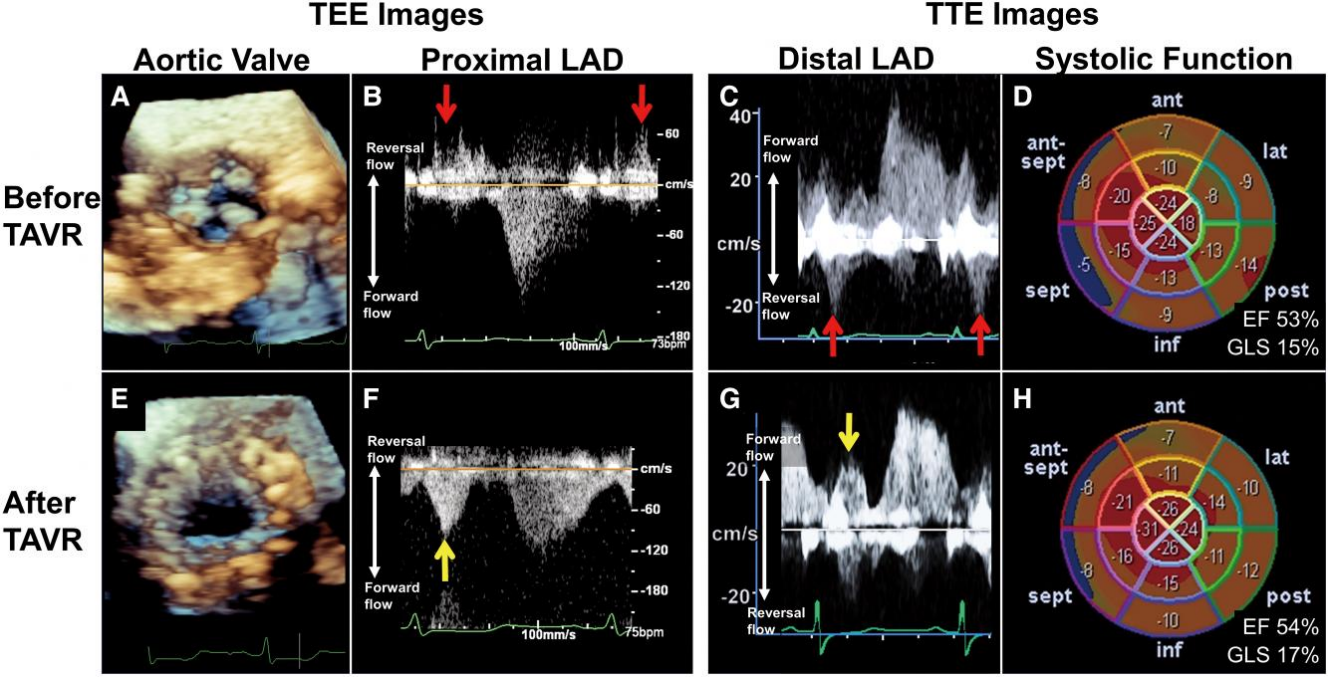
(*A*) The aortic cusp motion is severely restricted. (*B* and *C*) Transoesophageal and transthoracic echocardiography with pulse-wave Doppler scans show proximal and distal left anterior descending coronary artery flows, respectively. The left anterior descending coronary artery flow before transcatheter aortic valve replacement shows systolic coronary flow reversal (red arrows). (*D*) Transthoracic echocardiography scan before transcatheter aortic valve replacement shows a preserved left ventricular ejection fraction of 53% but a low endocardial global longitudinal strain of 15%. (*E*) A 26 mm self-expandable valve was successfully implanted, and the severe aortic stenosis was relieved. (*F* and *G*) Transoesophageal and transthoracic echocardiography scans after transcatheter aortic valve replacement show that the left anterior descending coronary artery flow in systole has changed from reverse to forward (yellow arrows). (*H*) Transthoracic echocardiography scan after transcatheter aortic valve replacement shows that endocardial global longitudinal strain has increased to 17%. EF, ejection fraction; GLS, global longitudinal strain; LAD, left anterior descending coronary artery; TAVR, transcatheter aortic valve replacement; TEE, transoesophageal echocardiography; TTE, transthoracic echocardiography.

A 79-year-old woman with severe AS and no coronary disease underwent transcatheter aortic valve replacement (TAVR). Preprocedural transthoracic echocardiography (TTE) showed a preserved left ventricular ejection fraction of 53% but a low endocardial global longitudinal strain (GLS) of 15% (*Figure 1D*). The distal left anterior descending coronary artery (LAD) flow showed systolic flow reversal (*Figure 1C*). Intraprocedural transoesophageal echocardiography (TEE) before valve implantation showed a severely calcified aortic valve (*Figure 1A*) and systolic flow reversal in the proximal LAD (*Figure 1B*). Following facility standards, a 26 mm self-expandable valve was implanted (*Figure 1E*). Intraprocedural TEE after valve implantation showed that the proximal LAD systolic flow changed from reverse to forward (*Figure 1F*). Postprocedural TTE revealed that the distal LAD systolic flow had changed from reverse to forward (*Figure 1G*; see Supplementary material online, *Video S1*) and GLS had increased to 17% (*Figure 1H*). The postoperative course was uneventful for 2 months.

Previous study has reported the dynamic changes in systolic coronary flow reversal during TAVR using TEE.^3^ Although assessing systolic and diastolic coronary flow in the same cardiac cycle using TTE is more challenging than using TEE, we observed a similar pattern to TEE in this case. The systolic flow reversal before TAVR may be explained by a high intraventricular systolic pressure that forces blood from within the endocardium back into the epicardial coronary arteries. And the restoration of coronary flow dynamics after TAVR might be associated with an increased endocardial GLS. Our findings may contribute to an understanding of the cardiac–coronary interactions in AS in daily clinical practice by simply recognizing coronary haemodynamics using TTE.

## Supplementary Material

ytae037_Supplementary_Data

## Data Availability

The data underlying this article will be shared upon reasonable request to the corresponding author.

## Supplementary material

**Consent:** The authors confirm that written consent for submission and publication of this case report, including images and associated text, has been obtained from the patient in line with COPE guidance.

**Funding:** None declared.
